# Transplacental Transfer of Hepatitis B Neutralizing Antibody during Pregnancy in an Animal Model: Implications for Newborn and Maternal Health

**DOI:** 10.1155/2014/159206

**Published:** 2014-03-27

**Authors:** Li Ma, Malgorzata G. Norton, Iftekhar Mahmood, Zhong Zhao, Lilin Zhong, Pei Zhang, Evi B. Struble

**Affiliations:** ^1^Laboratory of Plasma Derivatives, Division of Hematology, Office of Blood Research and Review, Center for Biologics Evaluation and Research, FDA 1401 Rockville Pike, Rockville, MD 20852, USA; ^2^Division of Hematology, Office of Blood Research and Review, Center for Biologics Evaluation and Research, FDA 1401 Rockville Pike, Rockville, MD 20852, USA

## Abstract

Despite the success of postexposure prophylaxis (PEP) of the newborn in preventing mother-to-child transmission of hepatitis B virus), in non-US clinical trials, administering hepatitis B immune globulin (HBIG) to mothers at the end of pregnancy (in addition to passive-active PEP of the newborn) only partially improved outcomes. That is, a significant percentage of newborns became infected during their first year of life. We used a relevant animal model for human IgG transplacental transfer to study dose, time and subclass dependence of HBV neutralizing antibody (nAb) maternal, and fetal levels at the end of pregnancy. Pregnant guinea pigs received 50 or 100 IU/kg HBIGIV 2–5 days before delivery. Human total IgG, IgG subclasses, and nAb in mothers and their litters were measured.* In vitro* analyses of guinea pig Fc neonatal receptor binding to HBIGIV, as well as to all human IgG subclasses, were also performed. Our study showed that nAb transferred transplacentally from the pregnant guinea pigs to their litters; no transfer occurred during parturition. The amount of the transferred nAb was dose and time dependent. Thus, selection of an efficacious dose in the clinic is important: microdosing may be underdosing, particularly in cases of high viraemia.

## 1. Introduction

Chronic hepatitis B is a serious viral disease, associated with a high risk for developing liver cirrhosis and hepatocellular carcinoma [1–3]. Worldwide, two billion people have been infected with the virus and about 600,000 people die every year due to the consequences of hepatitis B [4]. HBV is endemic in China and other parts of Asia where 8–10% of the adult population is chronically infected, often since birth. Because 90% of infants infected at birth develop chronic HBV infection and 25% of those die prematurely from cirrhosis or liver cancer [5, 6], failure to prevent infection following perinatal exposure to HBV carries a heavy burden to the individual, family, and society at large.

Passive-active postexposure prophylaxis (PEP) with HBIG and hepatitis B vaccine is 85–95% effective in preventing vertical transmission of HBV compared to 70–95% prevention rate for the vaccine alone [6, 7]. Despite PEP, 3–13% of infants born to infected mothers acquire HBV [8]. The risk is particularly high for children born to mothers with a high viral load [9, 10]. Although pregnancy, especially the peripartum period, has been associated with an increase in hepatitis B viral load [11], maternal use of antiviral drugs, such as nucleoside analogs, as an adjunct to PEP of the newborn has not shown conclusive benefit over PEP alone [12]. Similarly, using HBIG at the end of pregnancy has not yielded significant reductions in HBV neonatal infection rates of vaccinated babies by the time they reach one year of age [10]. Intrigued by these reports, we set out to characterize HBV neutralizing antibody levels and pharmacokinetic characteristics in the mother and her newborn, respectively, after HBIG administration in an animal model of pregnancy. Our data shows that neutralizing antibody in HBIG can pass the placenta in a dose and time dependent manner. This finding has implications when considering an efficacious dose in the clinic.

## 2. Materials and Methods

### 2.1. Animal Study

All animal procedures were performed in accordance with protocols approved by the CBER Animal Care and Use Committee. Hartley Albino (Crl:HA) guinea pigs were purchased from commercial sources. The animals were housed in pairs or individually, and food and water were provided* ad libitum*. Female guinea pigs were mated in accordance with a published protocol [13] to produce timed pregnancies and HBIGIV was administered as previously described [14]. The* in vivo* study is summarized in Table 1. Briefly, twelve pregnant guinea pigs at an average age of 185 days, weighing on average 1206 g received HBIGIV on GD 65–69 at 50 IU/kg (*n* = 6) or 100 IU/kg (*n* = 6). An additional six nonpregnant controls, at a mean age 177 days, weighing on average 862 g received the same doses (*n* = 3/dose group). Blood samples were collected by percutaneous femoral vein puncture at 10, 30 and 60 minutes and then every day until farrowing or termination. All pregnant guinea pigs gave birth 2–5 days after test article administration, except one in the high dose group which delivered within four hours after receiving HBIGIV and was excluded from analysis. There were a total of 34 live and 3 stillborn piglets born, at an average weight of 107 g/piglet. Terminal blood samples were collected via cardiac puncture under anesthesia.

### 2.2. Serum Processing and ELISA

Blood samples were stored overnight at 4°C to coagulate and then spun in a benchtop centrifuge at 1500 ×g for 5 minutes. Serum was collected, transferred into fresh tubes, and then frozen at −80°C for storage. Total IgG and neutralizing antibody levels were determined by using Human IgG ELISA Kit (Bethyl Laboratories, Montgomery, TX) and ETI-AB-AUK PLUS (DiaSorin, Saluggia, Italy), respectively. IgG subclasses were measured by using human IgG subclass kits (Invitrogen). Each sample was run in duplicate and a standard curve was included in each plate.

### 2.3. Pharmacokinetic Analysis

Pharmacokinetic parameters from plasma concentration-time data in pregnant and nonpregnant guinea pigs were estimated by noncompartmental analysis. Half-life was calculated by regression analysis on the terminal phase of concentration-time data. Clearance (CL) was estimated as follows:

(1) $$\begin{array}{c} \text{CL}=\frac{\text{Dose}}{\text{AUC}}, \end{array}$$

where AUC is the area under the curve calculated by the trapezoidal rule.

### 2.4. Guinea Pig FcRn and IgG Purification

A soluble version of human FcRn was constructed and purified as described [14]. Briefly, stably transfected Huh7 cells were grown in T-75 culture flasks (Fisher Scientific, Pittsburgh, PA) in GlutaMAX DMEM supplemented with 10% FBS until they reached confluence, at which point the medium was replaced with BD Cell MAB Serum Free Medium (Fisher) containing zeocin. The cultures were kept at 37°C in a CO_2_ incubator for two weeks, then the growth medium was collected and its pH adjusted to 6.0 with 1 M HCl. The solution was loaded by gravity flow onto a column of 1 mL IgG Sepharose 6 Fast Flow resin (GE Healthcare Life Sciences, USA) equilibrated with binding buffer containing 50 mM Na phosphate pH 6, 150 mM NaCl, and 0.005% Tween 20. After being washed with 3 mL binding buffer, the column was eluted with elution buffer containing 50 mM Tris Cl pH 8.5 and 150 mM NaCl. The eluate was analyzed by western blot and then concentrated via ultrafiltration with a 30 KDa cutoff device (Millipore).

IgG subclasses purified from myeloma were obtained commercially (Sigma, 1 mg/mL) and then dialyzed overnight into binding buffer in a dialysis cassette (3 mL capacity, 10,000 KDa cutoff, Pierce). The concentration was determined by measuring absorption at 280 nm with a spectrophotometer (Molecular Systems), using a molar absorption coefficient 2 × 10^5^ M^−1 ^cm^−1^. The samples were serially diluted threefold to obtain concentrations of 4000–48 nM which were then used in SPR measurements.

### 2.5. Surface Plasmon Resonance (SPR)

Binding assays were performed at 25°C using the Biacore 3000 instrument (GE Healthcare, Piscataway, NJ, USA). Guinea pig soluble FcRn (GPFcRN) diluted in 10 mM sodium acetate, pH 4.0 was immobilized on a CM5 chip using an amine coupling kit (GE Healthcare, Piscataway, NJ, USA) to a density of 1000 resonance units (RU). A reference surface was also created by performing the amine coupling protocol with buffer only. IgG subclasses were dialyzed into the binding buffer and serially diluted threefold to obtain concentrations of 4000–48 nM. The diluted IgG subclasses were injected in duplicate across the reference and GPFcRn surface at 50 *μ*L/min for 2 min. After dissociating for 3 min with the binding buffer, the chip surface was regenerated using elution buffer.

The binding responses were double referenced by subtracting the signals from the reference cell and buffer-only injections from the analyte injections. Estimated *K*_*D*_ values were derived by fitting the binding and dissociation signals with a 1 : 1 (Langmuir) model using the BIA evaluation 4.1.1 software.

### 2.6. Anti-Human Immune Response

ELISA was used to measure guinea pig anti-human antibody formation. For this, strips precoated with anti-human IgG (Bethyl Laboratories, Montgomery, TX) were incubated with a 1 : 1000 dilution of HBIGIV in super block blocking buffer (thermoscientific) at room temperature for 1 h. The plate was washed four times with phosphate-buffered saline (PBS; pH 7.4) with 0.05% Tween 20 to remove unbound human IgG. Guinea pig serum samples were added to the plate, followed by incubation at 37°C for 1 h. The plate was then washed four times before adding goat anti-guinea pig antibodies (heavy chain and light chain, 1 : 100 dilution) conjugated to horse radish peroxidase (Abcam Inc.) and then incubated at 37°C for 1 h. After four washes, the reaction was developed with 1-step TMB-ELISA substrate solution (KPL) and stopped by adding 100 *μ*L of 4 N sulfuric acid. The absorbance of each well was measured at 450 nm with a SpectraMax M2e microplate reader (Molecular Devices, Sunnyvale, CA).

### 2.7. Data Analysis

Absorbance values from ELISA were transformed into human antibody concentration or anti-HBs international units by fitting them to an equation derived from a five-parameter fit of the standard curve (SoftMax Pro). The values from all the siblings in each litter were averaged to obtain one single value. The fetal/maternal ratio for each litter was calculated by dividing the litter average by the concentration from its dam. This ratio for each litter over time was fitted using GraphPad Prism version 5.04 for Windows, GraphPad Software (San Diego, CA).

PK parameters from the pregnant and nonpregnant control guinea pigs were analyzed using unpaired, one tailed *t*-test and the *P* values reported; *P* values <0.05 were considered significant.

Differences in the mean nAb levels for the pregnant guinea pigs, nonpregnant controls, and piglets were analyzed using one way ANOVA with Bonferroni posttest for the high and low dose groups, respectively, using GraphPad Prism 5.04. *P* values <0.05 were considered significant.

## 3. Results

### 3.1. Human Antibody Transplacental Transfer

**Figure 1:**
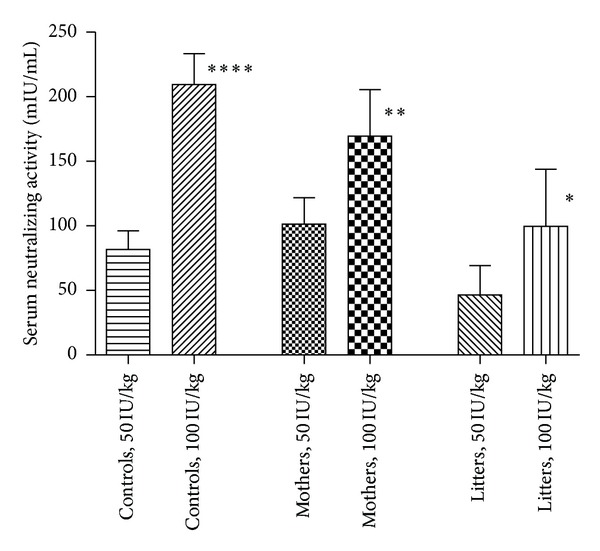
Neutralizing antibody in guinea pigs and their litters, averages group comparison.

There was antibody transfer from the pregnant guinea pigs to all the fetuses in both dose groups. One notable exception was the one pregnant which delivered within four hours after HBIGIV administration. None of the piglets in her litter had measurable human antibody in their serum, indicating that no appreciable transfer of IgG occurs during delivery. Both total human IgG and neutralizing IgG were detected in all the remaining guinea pigs and all the piglets in their litters at all the time points starting on day 2 (48 hours) after administration. The transfer of neutralizing antibody was dose dependent: the higher the administered dose is, the higher the average neutralizing antibody activity in the litter is (Table 2 and Figure 1). Regardless of the dose, the level of neutralizing antibody in all litters was higher than 10 mIU/mL, the accepted serological level for protection.

### 3.2. Human Antibody Transplacental Transfer Kinetics

**Figure 2:**
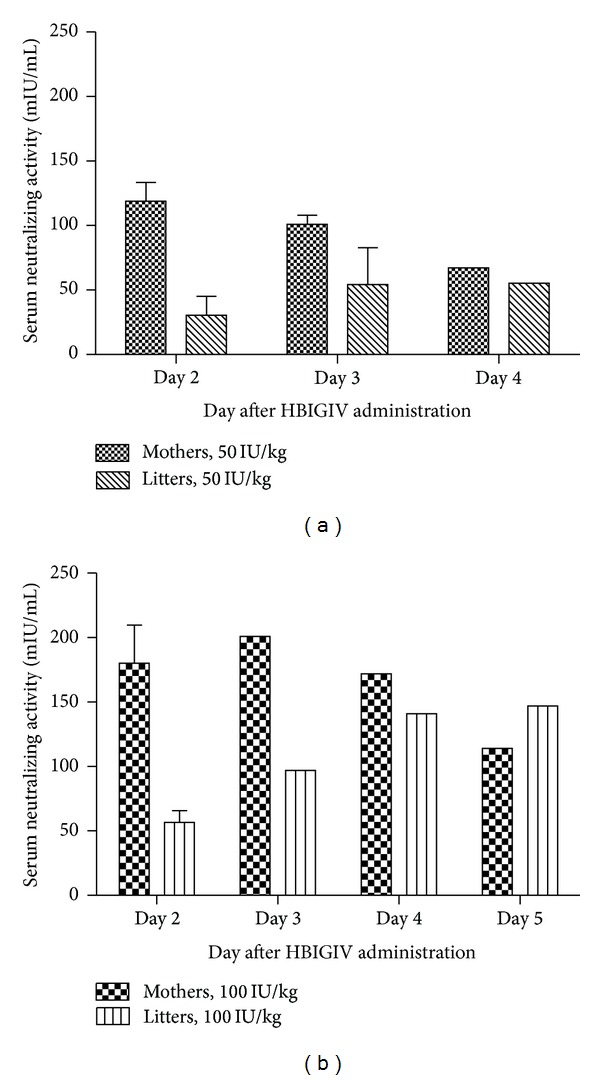
Neutralizing antibody in guinea pigs and their litters at 50 (a) and 100 IU/kg (b) dose.

**Figure 3:**
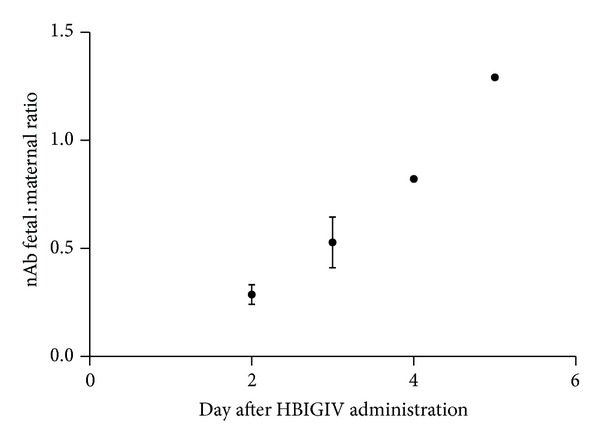
Kinetics of the neutralizing antibody fetal : maternal ratio.

As our preliminary data indicated [14], fetal concentration of neutralizing antibody increased with time. For each dose group, the litters born on day two had lower serum neutralizing activities than those born on subsequent days (Figure 2). The litter born five days after maternal HBIGIV administration had neutralizing antibody levels higher than their mothers'. The fetal : maternal ratio of the neutralizing antibody activity did not seem to depend on the dose and increased linearly with time in both dose groups (Figure 3). Based on a linear fit (*R* ^2^ = 0.7), the fetal :  maternal ratio increased at a rate of ~0.2/day. A similar value was observed when the ratios of total IgG concentrations were fitted (data not shown). The data also fits well using an exponential growth curve (*r* ^2^ = 0.8), perhaps indicating that FcRn expression levels may be increasing with gestation age.

### 3.3. IgG Subclass Transfer

IgG subclasses one through three were detected in all guinea pigs and litters. The amount of IgG4 in all animals, including nonpregnant controls, was below the detection limit because of the low concentration of IgG4 in the HBIGIV preparation used.

**Figure 4:**
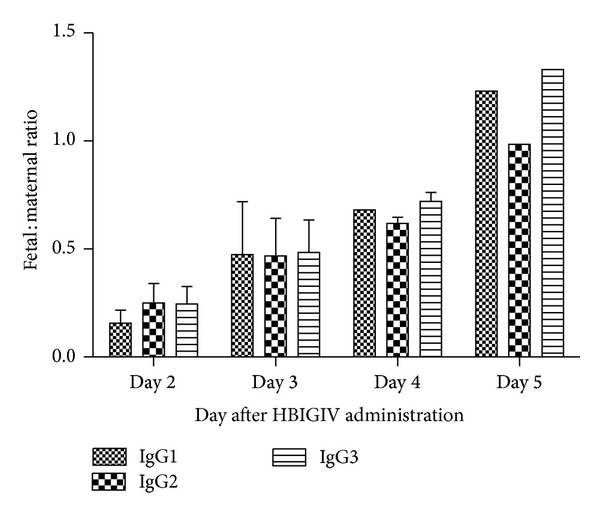
Kinetics of the fetal : maternal ratio for IgG subclasses.

The fetal : maternal ratio for the other three subclasses paralleled that of the total and neutralizing antibody and was comparable for all subclasses (Figure 4).

### 3.4. Anti-Human Antibody Formation

As expected, there was no guinea pig anti-human antibody formation up to day 5 following HBIGIV administration. Anti-human IgG antibodies were measureable on one nonpregnant control on days 14 and 21 after administration, demonstrating the ability of our test system to detect these antibodies (data not shown).

### 3.5. Pharmacokinetics of Human IgG in the Guinea Pig Model

Human IgG had different PK characteristics in pregnant guinea pigs compared to nonpregnant controls. On average, pregnant guinea pigs (Table 3) exhibited lower AUC, higher clearance (*P* = 0.06), and shorter half-life (*P* = 0.03) than the nonpregnant controls.

### 3.6. SPR of Guinea Pig FcRn Binding to Human IgG

**Figure 5:**
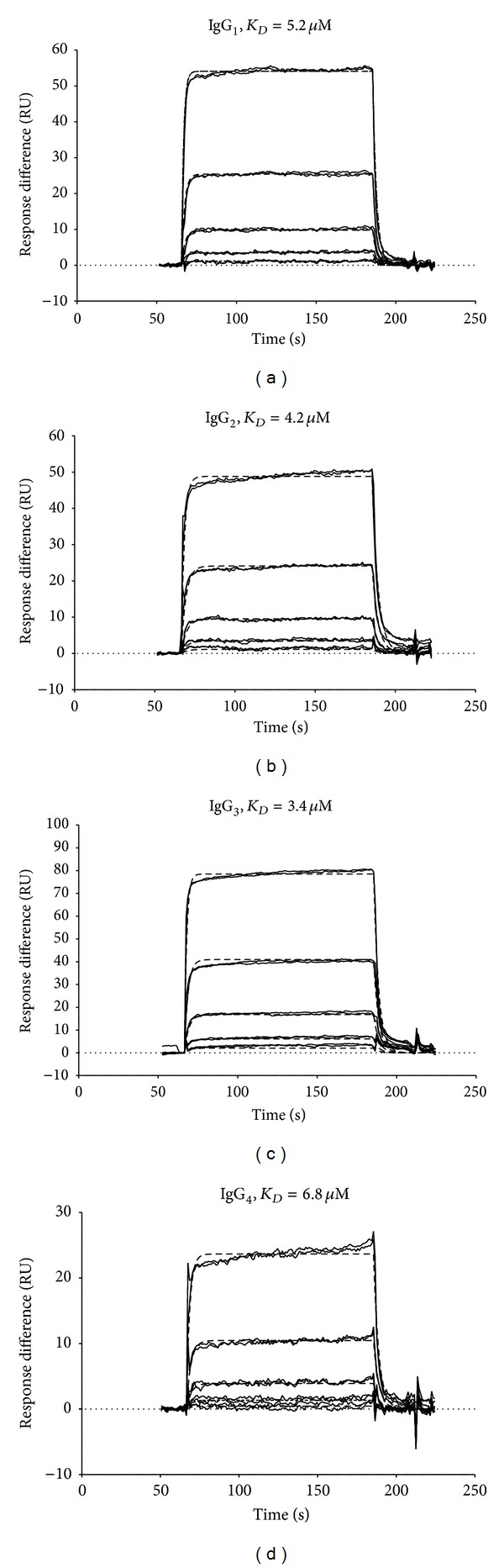
Binding of human IgG subclasses 1 through 4 to guinea pig FcRn receptor; panels (a) through (d), respectively. Dissociation constants (*K*_*D*_) range from 3.4 to 6.8 mM.

All human IgG subclasses bound to the receptor (Figures 5(a)–5(d)) with dissociation constants (*K*_*D*_) ranging from 3.4–6.8 *μ*M.

## 4. Discussion

For more than two decades, WHO and CDC have recommended universal hepatitis B vaccination for all newborns during the first year(s) of life. This strategy is yielding results: in USA, it has been credited with an estimated 81% decline in new HBV infections since 1991 when it was first implemented [15]. With the ever-increasing number of countries, including the endemic areas [4, 16, 17], incorporating HBV vaccination in their national infant immunization programs, the overall burden of the disease is expected to decrease in the coming years. However, one subset of the vaccines has not shared the benefits of this global success story. Despite PEP, a small but significant percentage of infants born to infected mothers still acquire HBV at birth [8]. The reasons for the prevention failure are not clear, but several factors play a role. For example, children of mothers who are HBeAg positive or with higher HBV DNA have significantly higher risk of becoming infected, showing that uncontrolled maternal infection is an important contributor [9]. Preterm infants weighing <2,000 g at birth do not respond well to the first dose of HBV vaccine given <24 hours after birth, indicating that immune immaturity may also be a contributing factor [18]. The two may also be linked, given that the risk of preterm labor is increased in women with exacerbation of chronic hepatitis B during pregnancy [12, 19].

If some or a combination of the enumerated factors are the cause for PEP failure, passive immune prophylaxis of the mothers with Hepatitis B Immune Globulin (i.e., HBIG and HBIGIV) would be beneficial. However, although HBIG has been used in pregnant women outside USA to help prevent vertical transmission of HBV, these studies have not shown clear efficacy.

Given the lack of data, we set out to gain a better understanding of the possible efficacious dose and kinetics of administered polyclonal antibody preparations during pregnancy. We administered two HBIGIV doses at the end of gestation in pregnant guinea pigs, both much higher than doses used in non-US clinical trials [10]. The low dose groups received 50 IU/kg HBIG, consistent with the label indication for prophylaxis following perinatal exposure [6]; the other dose was twice as high. Both doses resulted in neutralizing antibody concentrations in the newborns larger than 10 mIU/mL. Litters from the higher dose group had significantly more neutralizing antibodies than the lower dose group.

Newborn guinea pigs at birth had nAb levels that increased with the number of days from antibody administration to their mothers. Thus, for each dose group, litters born on day 2 had lower anti-HBs serum concentrations than those born on subsequent days (Figure 2). Consequently, each guinea pig litter essentially functioned as “a sink” for the human antibody product administered to their mother. This would imply altered pharmacokinetic parameters for HBIGIV during pregnancy in the guinea pig.

PK analysis of human IgG showed increased clearance and decreased AUC in pregnant compared to nonpregnant guinea pigs. The half-life was also decreased. Although statistically not significant, the difference in AUC between pregnant and nonpregnant guinea pigs indicates a physiological significance given the transplacental transfer of maternally administered antibodies to newborn guinea pigs.

The time-dependent accumulation of human IgG in the guinea pig fetal circulation at the end of pregnancy (Figures 2 and 3) is highly evocative of the increasing fetal : maternal IgG ratios derived from cord blood/maternal blood paired clinical samples [20–22].

A recent meta-analysis of Chinese clinical studies demonstrated that administering multiple small doses of HBIG, typically 200 IU on weeks 28, 32 and 36 of pregnancy, decreases the rate of infection for newborns [10]. A single 200 IU HBIG dose, depending on the specific lot, corresponds to a dose volume of 0.64 mL or less, and, for a 75 kg pregnant woman would be at least 7 times lower than 0.06 mL/kg, the recommended dose for post exposure prophylaxis. Thus, we refer to this dose as a “microdose”. Interestingly, among infants for whom anti-HBs data was collected, a larger percentage had neutralizing antibodies after microdosing their mothers with HBIG* versus* controls, also demonstrating (as did our guinea pig studies) that nAbs in HBIG cross the placenta to reach pharmacologically active levels in the fetus.

Although this recent meta-analysis [10] adds strong support to the benefit of passive immunization strategies to help prevention of HBV vertical transmission before birth, the odds ratios are consistent with treatment failure for many infants. Our studies in the guinea pig prompt us to posit that maternal microdosing may not afford an adequate and sustained increase in maternal anti-Hbs levels. Given the dose-dependent nAb transplacental transfer we observed in our animal model, these lower maternal nAb would result in lower fetal concentrations. Furthermore, antibody pharmacokinetics in pregnant chronically infected women may be different from what we saw in our animal model in the absence of infection. For example, in women with high viraemia or those experiencing pregnancy related HBV exacerbations, increased antibody clearance via antibody “sequestration” through opsonization, as seen in other antibody antiviral treatments [23, 24], could be occurring, thus further decreasing maternal (and fetal) neutralizing antibody levels. Our data leads us to believe that microdosing with HBIG during pregnancy may not be sufficient and, with or without exacerbations, higher or more frequent dosing at the end of gestation could improve outcomes for the newborns.

As with all antibody therapies during pregnancy, the risk for antibody facilitated, FcRn mediated transplacental viral transmission is a possibility. A recent clinical trial in HIV infected mothers receiving one dose of HIV hyperimmune globulin preparation during gestation weeks 36–38 considered but discounted its likelihood for HIV [25]. In the case of HBV, the odds ratios at birth favor HBIG usage during pregnancy [10], thus too excluding the possibility for antibody-mediated increased transmission.

It should be stressed that our study has inherent limitations. By using the pregnant guinea pig, a good model for transplacental transfer of human antibody, our study in guinea pigs does not account for HBV infection. It also is limited by a small sample size: twelve pregnant and six nonpregnant control guinea pigs, with one guinea pig and her litter excluded from analysis due to delivering within four hours of HBIGIV injection. Although transplacental transfer of nAb was evident in all the litters born two or more days after HBIGIV administration, in a statistically significant dose dependent manner, it is currently unknown if the same observation could be made in infected pregnant women. Despite being informative, animal studies such as this are not a replacement for clinical studies. The safety and efficacy of antibody preparations, including HBIG when used to prevent vertical transmission of HBV during pregnancy, will ultimately be determined in well-designed, adequately powered, randomized, controlled clinical studies.

## Figures and Tables

**Table 1:** Summary of the *in vivo* study.

	Number of animals	Mean weight (range), g	Dose, IU/kg		Exposure, days	Mean litter size (range)	Mean litter weight (range), g
Pregnant	12	1206 (920–1360)	50 (*n* = 6)	100 (*n* = 6)	2–5	3 (1–5)	107 (82–150)

Nonpregnant	6	862 (764–924)	50 (*n* = 3)	100 (*n* = 3)	5	N/A	N/A

**Table 2:** Serum neutralizing antibody levels (mIU/mL).

Dose	Nonpregnant controls (range)	Mothers (range)	Litter (range)
50 IU/kg	82 (67–96)****	101 (67–129)**	47 (11–110)*
100 IU/kg	209 (183–231)****	169 (114–201)**	90 (50–141)*

****P < 0.0001; **P < 0.01; *P < 0.05; *P* values were derived from *posthoc* comparisons between 50 and 100 IU/kg groups in each of the cohorts, respectively.

**Table 3:** Average pharmacokinetic parameters for human IgG (50 IU/kg dose) in the pregnant guinea pigs and the nonpregnant controls.

	AUC^b^ (*μ*g∗hr/mL)	CL^c^ (mL/hr per kg)	Half-life (hours)
Pregnant, mean (STDEV)^a^	1819 (501)	1.396 (0.381)	59 (25)*

Nonpregnant, mean (STDEV)	2770 (846)	0.916 (0.245)	95 (4)*

*P < 0.05.

^a^One pregnant guinea pig was excluded from analysis due to abnormally high AUC (4-5 times higher than the other sows).

^b^Area under the curve.

^c^Clearance.

## Abbreviations

HBV: Hepatitis B virus
HBIG: Hepatitis B immune globulin
Anti-HBs: Antihepatitis B surface antigen
HBeAg: Hepatitis B e-antigen
PEP: Postexposure prophylaxis
HBIGIV: Hepatitis B immune globulin intravenous (human)
nAb: Neutralizing antibody
SPR: Surface plasmon resonance
RU: Relative units
CL: Clearance
AUC: Area under the curve
MTCT: Mother-to-child transmission.
